# Case Report: Purtscher-like retinopathy in a patient with lung adenocarcinoma

**DOI:** 10.12688/f1000research.74917.1

**Published:** 2022-02-24

**Authors:** Anis Mahmoud, Asma zaghdoudi, Soumaya Boucharb, Fatma Abid, Sameh Mbarek, Hassan Ibn Hadj Amor, Sawssen Braiek, Nadia Keskes Boudawara, Jalel knani, Riadh Messaoud

**Affiliations:** 1 ophtalmology, Taher Sfar University Hospital, Mahdia, Mahdia, 5100, Tunisia; 2 Faculty of Medicine, University of Monastir, Monastir, Tunisia; 3 Pneumology, Taher Sfar University Hospital, Mahdia, Mahdia, 5100, Tunisia; 4 Cardiology, Tahar Sfar University Hospital, Mahdia, Mahdia, 5100, Tunisia; 5 Carcinology, Tahar Sfar university Hospital, Mahdia, Mahdia, 5100, Tunisia

**Keywords:** Purtscher-Like Retinopathy, Lung Adenocarcinoma, corticosteroids

## Abstract

This case report describes an unreported case of  Purtscher-like retinopathy in a patient with pulmonary adenocarcinoma. A 39-year-old man was hospitalized for exploration of a hemoptysis and bilateral blurry vision. Fundoscopic examination showed multiple areas of retinal whitening in the peripapillary area. A chest computed tomography scan then showed a ground glass opacity in the right upper lobe associated to a hilar lymphadenopathy.

A thoracotomy and lung biopsy were performed concluding with the diagnosis of lung adenocarcinoma. The patient was treated with high-dose corticosteroids and received Taxol-Carboplatin chemotherapy with good visual outcomes.

The article discusses furthermore the importance of including pulmonary adenocarcinoma to the list of systemic conditions for Purtscher-like retinopathy.

## Introduction

Purtscher's retinopathy is a rare condition occurring in the context of trauma, and it was first described by Otmmar Purtscher in 1910. ^1^ Patients with similar retinal findings, such as bilateral cotton wool spots and haemorhages in non-traumatic circumstances are labeled “Purtscher-like” retinopathy. ^2^

We herein describe a previously unreported association of Purtscher-like retinopathy with lung adenocarcinoma. We aim to highlight the importance of identifying this clinical entity and to provide a credible hypothesis about inherent mechanism of this disease.

## Case report 

A 39-year-old Tunisian, unemployed man with no previous pathological history presented to the emergency department for exploration of recurrent hemoptysis.

**Figure 1.**
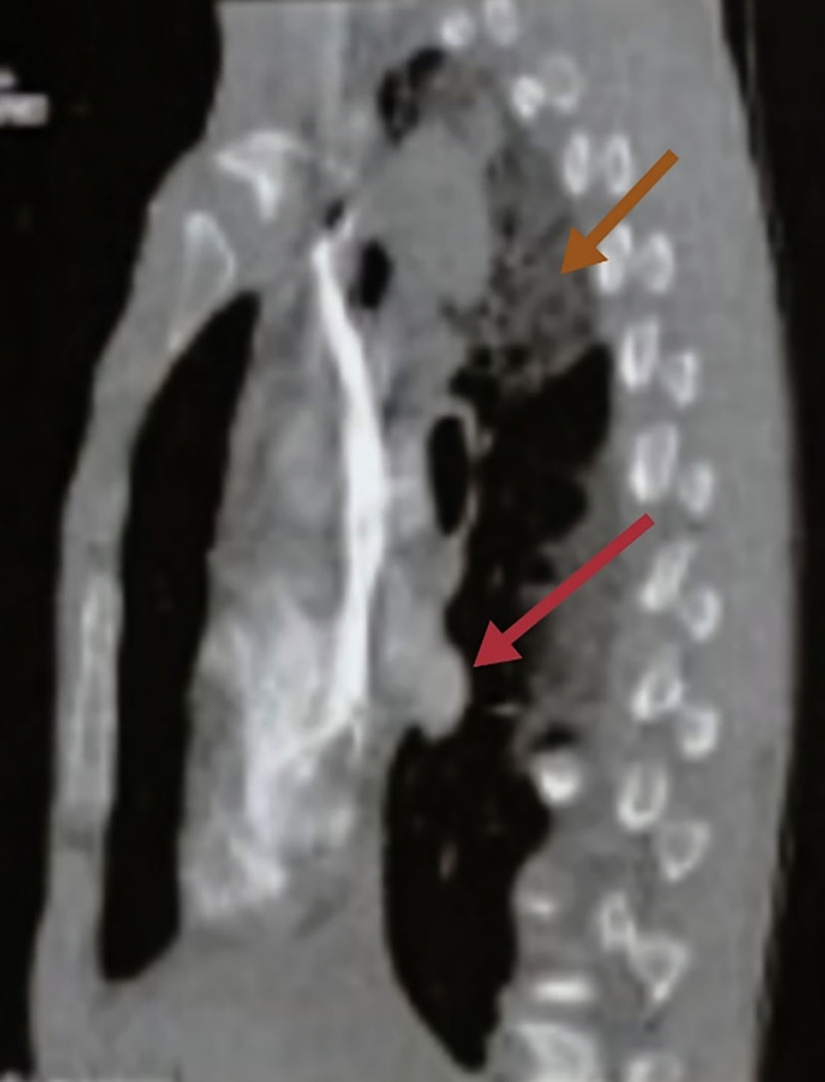
Chest computed tomography scan showed a ground glass opacity in the right upper lobe (brown arrows) associated to a hilar lymphadenopathy with irregular contours (red arrows).

A chest computed tomography (CT) scan revealed ground glass opacity in the right upper lobe, associated to a hilar lymphadenopathy measuring 42 mm*32 mm*28 mm with irregular contours and compressing the esophagus [ Figure 1].

Based on these results, the patient was admitted to the pulmonary department for further investigation and appropriate treatment including blood transfusion and hydration. Then, he was referred to the ophthalmology department to examine blurred vision present since the onset of his respiratory symptoms.

**Figure 2.**
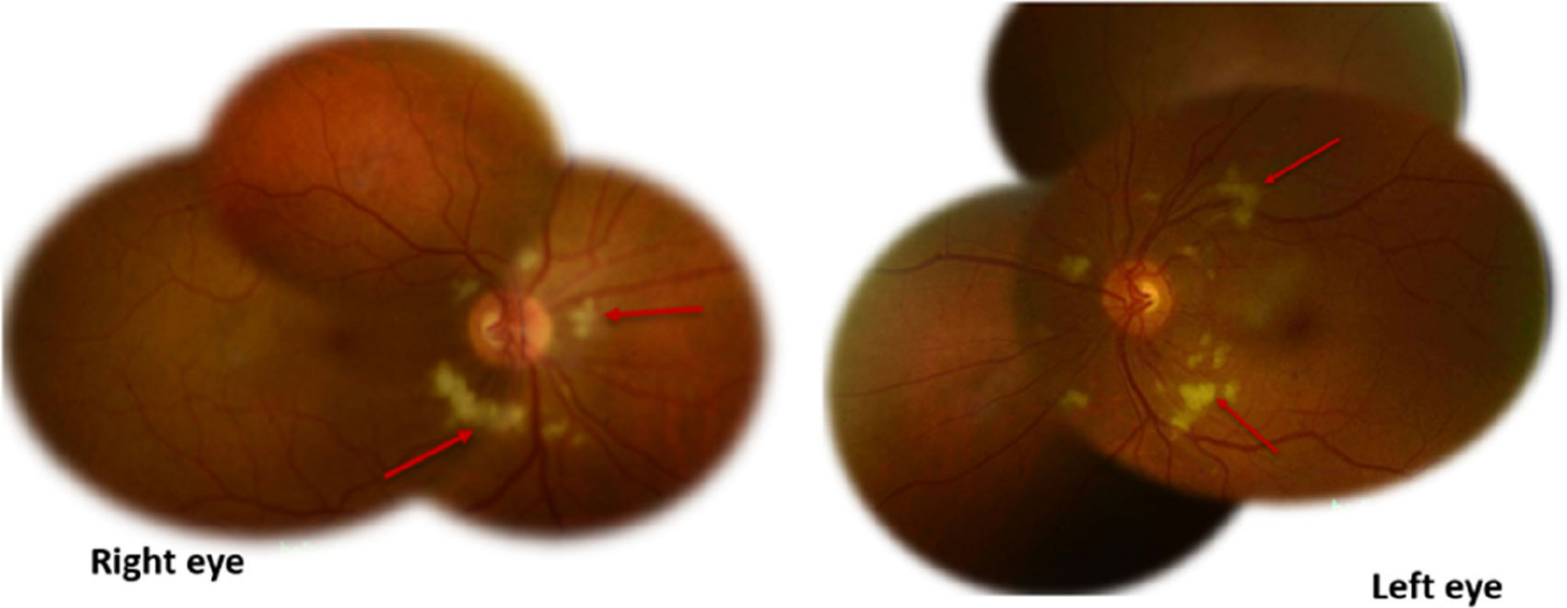
Composite fundus photographs showed multiple areas of retinal whitening in the peripapillary area (red arrows).

On examination, visual acuity was 20/40 in both eyes. The intraocular pressure was 12 mm Hg in both eyes (normal range, 10-21 mm Hg) and bilateral anterior segment examination showed no abnormalities. No defects in pupillary reflexes or eye movements were noted. Dilated fundus examination showed bilateral multiple areas of retinal whitening in the peripapillary area, resembling cotton wool spots [ Figure 2].

**Figure 3.**
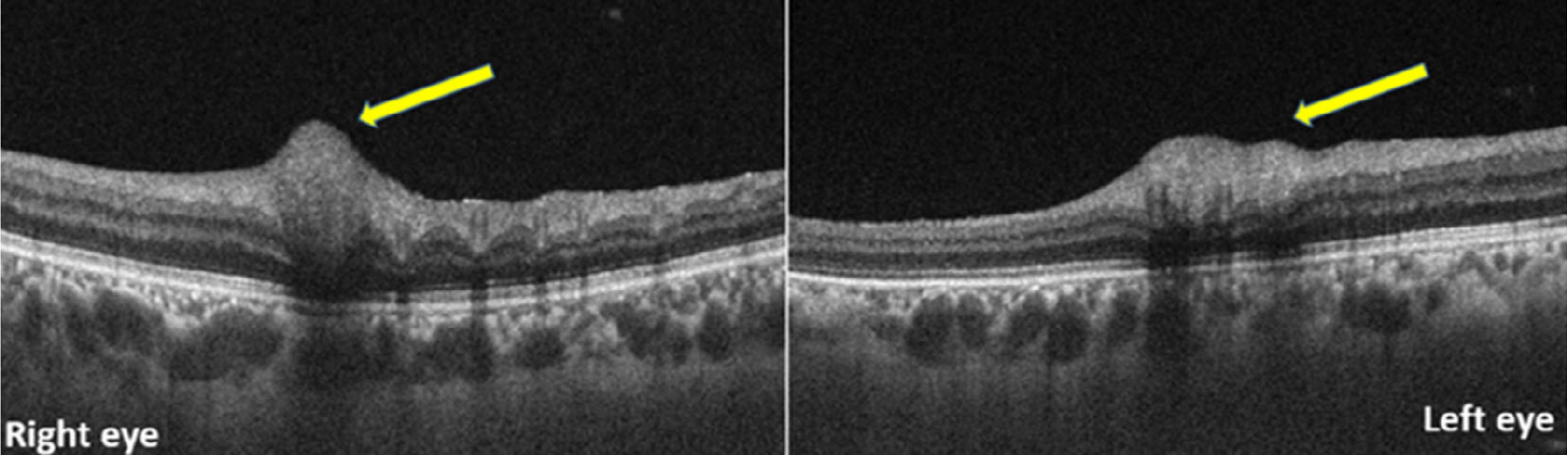
Optical coherence tomography passing through cotton wool spots revealed focal hyperreflectivity in the inner/middle retinal layers (yellow arrows).

Swept source optical coherence tomography scans passing through the cotton wool spots showed focal hyperreflectivity in the inner/middle retinal layers [ Figure 3].

**Figure 4.**
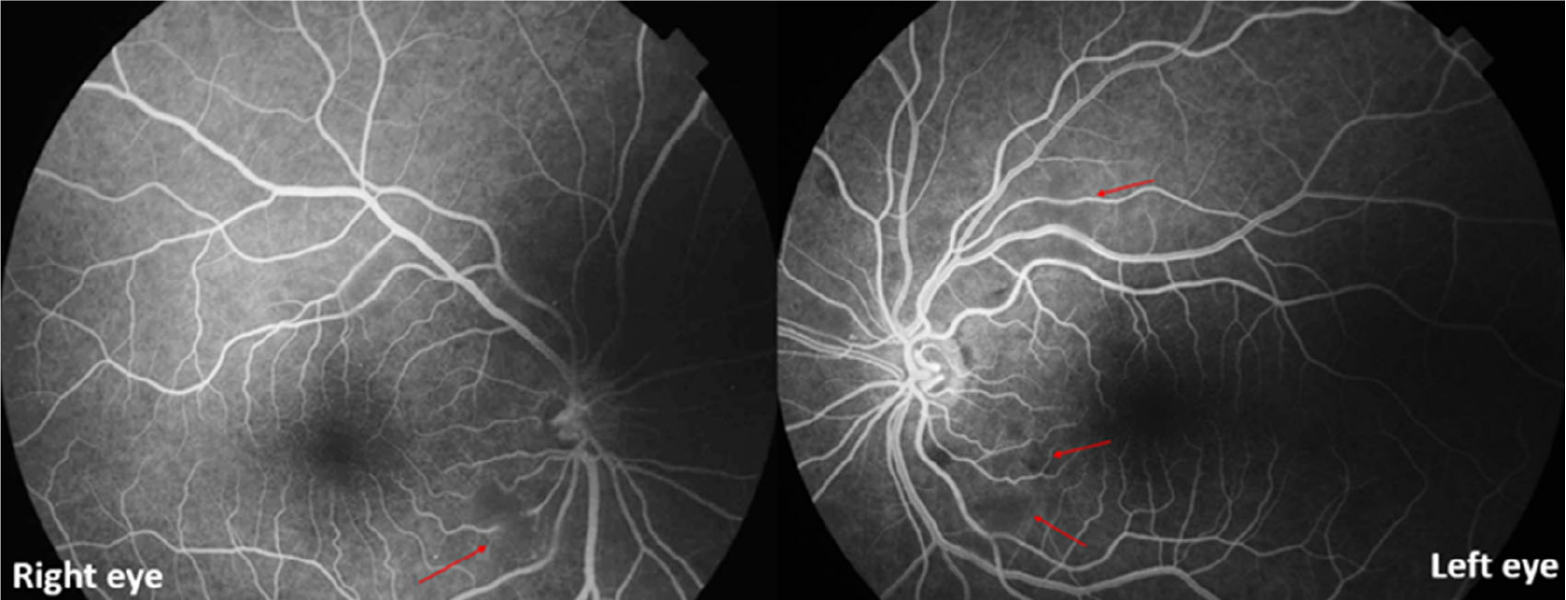
Fluorescein angiography demonstrated mild obscuration of the retinal vasculature in areas of cotton wool spots (red arrows).

The fluorescein angiography was unremarkable, except for mild obscuration of the retinal vasculature corresponding to the areas of cotton wool spots (red arrows) [ Figure 4].

On the basis of fundoscopic findings showing bilateral cotton wool spots limited to the peripapillary area in a patient with no traumatic background, the diagnosis of Purtscher-like retinopathy was made.

A thoracotomy and right upper lobe biopsy were performed leading to the conclusion and diagnosis of lung adenocarcinoma.

The patient was treated with high-dose intravenous methylprednisolone (1000 mg daily) for three days, followed by oral corticosteroids at a dose of 1 mg/kg/day with progressive degression over two months and received four courses of 200 mg/m ^2^ Taxol and Carboplatin at AUC of 6 mg/mL/min every 3 weeks. No chemotherapy-related adverse effects were noted. Following this treatment, the patient experienced significant clinical improvement and the hemoptysis resolved. A recent chest CT scan showed no remaining masses.

**Figure 5.**
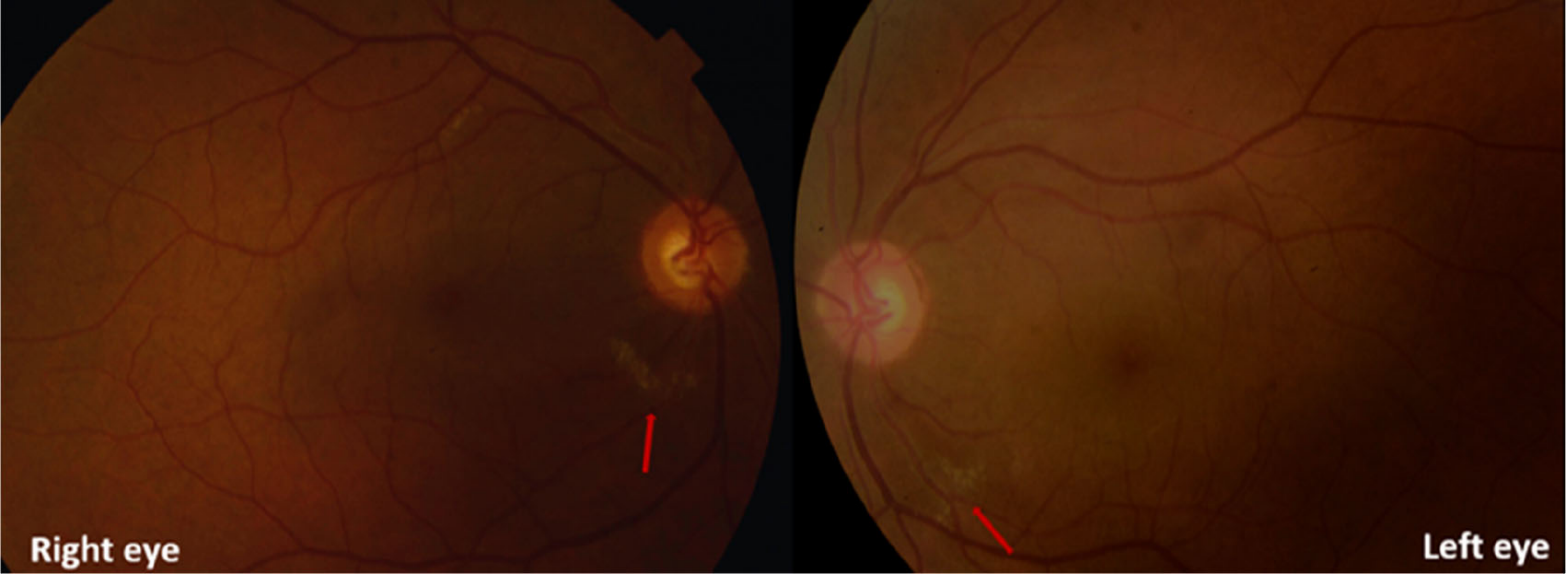
Fundus photographs showed decrease in the number and size of cotton wool spots (red arrows) in both eyes at 1-month follow-up.

On the ophthalmic side, the patient's visual acuity improved to 20/20. On dilated fundus examination [ Figure 5], there was significant decrease in the number and size of cotton wool spots in both eyes.

## Discussion 

Purtscher’s retinopathy is an occlusive microvasculopathy, which was first reported by Otmar Purtscher in 1910, in a patient with blanching whitening and hemorrhages at the posterior pole following severe head trauma. ^1^

Purtscher-like retinopathy includes all retinal lesions similar to the description by Otmar Purtshcer in 1910, ^1^ but occurring in non-traumatic context such as patients with acute pancreatitis, connective tissue disorders and lymphoproliferative diseases. ^2^

Both Purtscher retinopathy and Purtscher-like retinopathy are considered as extremely rare diseases, with a reported combined incidence rate of 0.24 per million. ^3^

Our patient’s ocular findings were bilateral cotton wool spots limited to the peripapillary area. Clinical course and favorable evolution following chemotherapy, appear to be consistent with Purtscher-like retinopathy complicating lung adenocarcinoma.

The exact pathogenesis of Purtscher and Purtscher-like retinopathy remains controversial. The theory most supported regarding its mechanism is attributed to an embolic occlusion involving the precapillary arterioles. ^4^

In malignancies, Purtscher-like retinopathy is considered as paraneoplastic disorder in which interactions of tumor cells with vascular endothelium, blood coagulation agents, and platelets contribute to its pathogenesis. ^5^^–^^7^

Lung adenocarcinoma is a cause of hypercoagulability, ^8^ and numerous reports highlight its association with retinal vascular thromboembolic events.

Ronchetto ^9^ described the case of a patient with branch retinal vein occlusion associated with lung carcinoma, and suggested that the retinal microangiopathic changes may result from the hypercoagulable state seen in such a tumor.

Madabhavi *et al* ^10^ described a central retinal artery occlusion during the evolution of a pulmonary adenocarcinoma. This incident was attributed to the stimulation of the coagulation activation system by tumoral cells.

Treatment of the acute phase of Purtscher’s retinopathy is based on high-dose intravenous corticosteroids, and visual recovery is variable, being directly related to the extent of non-perfusion areas on retinal angiography. ^11^ The visual outcome in our patient was good, which is consistent with the absence of extensive areas of non-perfusion on retinal angiography.

This case highlights the importance of ophthalmologic examination in patients with lung adenocarcinoma for suggestive findings of Purtcher-like retinopathy. Nevertheless, this association remains poorly explained and requires further documented cases.

## Conclusion

To the best of our knowledge, this case report is the first to describe the association between lung adenocarcinoma and Purtscher-like retinopathy and the mechanism of this association remains speculative. Despite this, we suggest that lung adenocarcinoma should be included in the list of systemic conditions of Purtscher retinopathy.

## Consent

The patient provided informed written consent for the publication of this case report and associated figures.

## Data availability

All data underlying the results are available as part of the article and no additional source data are required.
