# Supplementary material for: Case Report: Purtscher-like retinopathy in a patient with lung adenocarcinoma
Source: F1000Res. 2022 Feb 24;11:227. [Version 1] doi: 10.12688/f1000research.74917.1 (PMC8941289; doi:10.12688/f1000research.74917.1)
Supplement: Peer review history

# Peer review: Reviewer response for version 1

**DOI:** 10.5256/f1000research.78721.r125326

**Published:** 2022-03-22

**Authors:** Najeh Hcini

**Affiliations:** 1 Department of Obstetrics and Gynaecology, West French Guiana Hospital Center, Cayenne, France

The authors have written an interesting case report of a young person who developed Purtscher-like retinopathy in association with lung adenocarcinoma.

 The authors also explained well the possible pathophysiology of this entity as well as the risk of retinal vascular thrombosis associated with lung adenocarcinoma.

 The discussion section is well written and organised, which is sure to attract readers.

**Conclusion:**

 "To the best of our knowledge": please remove this phrase.

 "Despite this": please rephrase the last sentence.

 In total: Overall, I found this case report interesting and approve it for indexing.

Are enough details provided of any physical examination and diagnostic tests, treatment given and outcomes?

Yes

Is the case presented with sufficient detail to be useful for other practitioners?

Yes

Is sufficient discussion included of the importance of the findings and their relevance to future understanding of disease processes, diagnosis or treatment?

Yes

Is the background of the case’s history and progression described in sufficient detail?

Yes

Reviewer Expertise:

infectious diseases - immunity -Virus

I confirm that I have read this submission and believe that I have an appropriate level of expertise to confirm that it is of an acceptable scientific standard.

# Peer review: Reviewer response for version 1

**DOI:** 10.5256/f1000research.78721.r125328

**Published:** 2022-03-22

**Authors:** Meriem Ouederni

**Affiliations:** 1 Department of Ophthalmology, Habib Thameur Hospital, Tunis, Tunisia

The article describes an interesting unreported case of Purtscher-like retinopathy in a patient with pulmonary adenocarcinoma.

 Complete illustration of the case was provided. The case adds a new systemic condition associated with Putscher-like retinopathy.

 In the discussion section, pathogenesis was discussed even if the association remained poorly explained. Further documented cases are needed for better understanding of the relationship.

Are enough details provided of any physical examination and diagnostic tests, treatment given and outcomes?

Yes

Is the case presented with sufficient detail to be useful for other practitioners?

Yes

Is sufficient discussion included of the importance of the findings and their relevance to future understanding of disease processes, diagnosis or treatment?

Yes

Is the background of the case’s history and progression described in sufficient detail?

Yes

Reviewer Expertise:

medical retina, cornea, cataract

I confirm that I have read this submission and believe that I have an appropriate level of expertise to confirm that it is of an acceptable scientific standard.

# Peer review: Reviewer response for version 1

**DOI:** 10.5256/f1000research.78721.r125329

**Published:** 2022-03-17

**Authors:** Ayman G. Elnahry

**Affiliations:** 1 Department of Ophthalmology, Faculty of Medicine, Cairo University, Cairo, Egypt

This report describes a rare case of Purtscher-like retinopathy associated with lung adenocarcinoma. The article is well written and well illustrated and adds to the existing body of literature on associations with Purtscher-like retinopathy. It is interesting to elaborate on the possible association between increased intrathoracic pressure with the coughing associated with hemoptysis/valsulva (if it was excessive in this case) in relation to Purtscher-like retinopathy since chest trauma is a known cause of Purtscher retinopathy which may occur through increased intrathoracic pressure following the chest trauma leading to impaired venous drainage from the eye and stasis with resultant hemorrhage and CWS.

Are enough details provided of any physical examination and diagnostic tests, treatment given and outcomes?

Yes

Is the case presented with sufficient detail to be useful for other practitioners?

Yes

Is sufficient discussion included of the importance of the findings and their relevance to future understanding of disease processes, diagnosis or treatment?

Yes

Is the background of the case’s history and progression described in sufficient detail?

Yes

Reviewer Expertise:

Vitreoretinal disease.

I confirm that I have read this submission and believe that I have an appropriate level of expertise to confirm that it is of an acceptable scientific standard.
